# The validity of self-reported cancer screening history and the role of social disadvantage in Ontario, Canada

**DOI:** 10.1186/s12889-015-1441-y

**Published:** 2015-01-29

**Authors:** Aisha Lofters, Mandana Vahabi, Richard H Glazier

**Affiliations:** St. Michael’s Hospital Department of Family and Community Medicine, University of Toronto, Toronto, Canada; Centre for Research on Inner City Health, Li Ka Shing Knowledge Institute, St. Michael’s Hospital, Toronto, Canada; Institute for Clinical Evaluative Sciences, Toronto, Canada; Faculty of Community Services, Daphne Cockwell School of Nursing, Ryerson University, Toronto, Canada

**Keywords:** Self-report, Validity, Cancer screening, Socioeconomic status

## Abstract

**Background:**

Self-report may not be an accurate method of determining cervical, breast and colorectal cancer screening rates due to recall, acquiescence and social desirability biases, particularly for certain sociodemographic groups. Therefore, the aims of this study were to determine the validity of self-report of cancer screening in Ontario, Canada, both for people in the general population and for socially disadvantaged groups based on immigrant status, ethnicity, education, income, language ability, self-rated health, employment status, age category (for cervical cancer screening), and gender (for fecal occult blood testing).

**Methods:**

We linked multiple data sources for this study, including the Canadian Community Health Survey and provincial-level health databases. Using administrative data as our gold standard, we calculated validity measures for self-report (i.e. sensitivity, specificity, positive and negative likelihood ratios, positive and negative predictive values), calculated report-to-record ratios, and conducted a multivariable regression analysis to determine which characteristics were independently associated with over-reporting of screening.

**Results:**

Specificity was less than 70% overall and for all subgroups for cervical and breast cancer screening, and sensitivity was lower than 80% overall and for all subgroups for fecal occult blood testing FOBT. Report-to-record ratios were persistently significantly greater than 1 across all cancer screening types, highest for the FOBT group: 1.246 [1.189-1.306]. Regression analyses showed no consistent patterns, but sociodemographic characteristics were associated with over-reporting for each screening type.

**Conclusions:**

We have found that in Ontario, as in other jurisdictions, there is a pervasive tendency for people to over-report their cancer screening histories. Sociodemographic status also appears to influence over-reporting. Public health practitioners and policymakers need to be aware of the limitations of self-report and adjust their methods and interpretations accordingly.

## Background

Screening for cervical, breast and colorectal cancer (CRC) using the Papanicolaou (Pap) test, mammography, and the fecal occult blood test (FOBT) respectively are commonly accepted practices in primary care in Canada. Because of the effectiveness of the Pap test and its widespread use, Canada currently has one of the world’s lowest annual incidence and mortality rates for invasive cervical cancer [1-3]. Mammography is associated with significant reductions in the relative risk of death from breast cancer and has long been recommended by the Canadian Task Force on Preventive Health Care [4]. Colorectal screening rates in Canada are currently low, but it has been estimated that if 70% of the eligible population participated in screening, mortality due to CRC could drop by 13-15% [5].

Despite the known benefits of screening, the literature suggests that people who are members of certain sociodemographic groups are subject to cancer screening inequities, particularly ethnoracial minorities, immigrants, people with low levels of education, people with complex medical conditions, and those of low income [6-12]. The benefits of screening combined with the apparent inequalities in screening based on social disadvantage demonstrate the need for valid methods of determining and monitoring screening rates. It is therefore concerning to consider that self-report, a commonly used method for determining screening history, may have validity issues and that people from certain sociodemographic groups might be more likely to inaccurately report cancer screening than their peers [13-16]. Under-estimating screening prevalence or over-estimating screening inequalities could lead to wasted resources, and conversely, over-estimating screening prevalence or under-estimating screening inequalities could lead to missed opportunities for improving screening. Self-report is potentially vulnerable to acquiescence bias (the tendency to give positive responses to questions when in doubt) and social desirability bias (the tendency to respond in a manner that respondents believe will be viewed favourably), and both types of bias may be more common among socially disadvantaged groups [14,16,17].

Literature from the US suggests that these biases in self-report might exist differentially, and that Hispanics and African-Americans may be more likely to over-report screening [14,15]. However, little is known about the validity of self-report of cancer screening in Canadian populations or among particular Canadian sociodemographic groups. Therefore, the objectives of this study were: i) to determine the validity of self-report of cervical, breast and CRC screening in Ontario for people in the general population, and ii) to determine the validity of self-report for socially disadvantaged groups based on: immigrant status, ethnicity, education, income, language ability, self-reported health, employment status, age category (for cervical cancer screening), and gender (for CRC screening).

## Methods

### Data sources

Multiple data sources were used for this study. The Canadian Community Health Survey (CCHS) is a national, biennial, cross-sectional survey conducted by Statistics Canada that collects information related to health status and health care utilization, including questions on cervical, breast, and CRC screening, as well as demographic characteristics such as visible minority status, immigrant status, language ability, education, age and gender. The CCHS data to which we had access contain responses from approximately 30,000 Ontarians (approximately 60% of Ontario respondents) per cycle who agreed to data linkage with administrative health data and whose data were able to be linked. We used data from four cycles of CCHS data (2000–2001, 2003, 2005, 2007). The Ontario Health Insurance Plan (OHIP) Claims’ Database contains claims for physician and hospital services as part of the provincial universal health insurance plan and includes approximately 95% of physician claims in the province [18]. The Canadian Institute of Health Information Discharge Abstract Database consists of fee codes and diagnostic codes claimed by Ontario physicians for hospital stays and procedures. Cytobase is Ontario’s electronic Pap test registry. Finally, the Ontario Breast Screening Program data record the date of mammogram for all women who participate in the provincial screening program. Using a unique encoded identifying number that allows individuals to be tracked through multiple databases, we linked respondents from the four cycles of CCHS with the aforementioned health databases. This study was approved by the Research Ethics Board of Sunnybrook Health Sciences Centre.

### Inclusion/exclusion criteria

We created three groups in this study. Survey respondents were included if they had answered the questions about cancer screening and were eligible for cervical cancer screening (cervix group), breast cancer screening (breast group), or fecal occult blood testing (FOBT group) respectively. Cervical cancer screening eligibility consisted of being 24 to 69 years of age on the date that they completed the CCHS, being continuously eligible for health insurance coverage for the three years prior to the CCHS interview, no history of hysterectomy, and no previous diagnosis of cervical cancer. Similarly, breast cancer screening eligibility consisted of being 52 to 69 years of age on the date that they completed the CCHS, being continuously eligible for Ontario’s universal health insurance coverage for the two years prior to the CCHS interview, and no previous diagnosis of breast cancer. Women were excluded if they reported on the CCHS that they had a mammogram for diagnostic (as opposed to screening) purposes. Eligibility for screening with FOBT was defined as being 52 to 74 years of age on the date of the CCHS, being continuously eligible for health insurance coverage for the two years prior to the CCHS interview, no history of barium enema or sigmoidoscopy in the preceding five years, no history of colonoscopy in the preceding ten years, and no previous diagnosis of CRC. People were excluded if they reported on the CCHS that they had an FOBT as follow-up of a problem or follow-up of CRC treatment. Ages and time periods for each cancer screening type were determined by provincial guidelines [19-21].

### Validity measures

We calculated validity measures i.e. sensitivity, specificity, positive and negative likelihood ratios, positive and negative predictive values, and simple kappa statistics for each cancer screening type. A kappa of 0.6 to 0.79 indicates substantial agreement, whereas a kappa of 0.4 to 0.59 indicates only moderate agreement [22]. Specifically, we assessed if those eligible self-reported or had a record of a Pap test within the three years prior to the CCHS, a mammogram within two years, and FOBT within the two years prior to the CCHS. A record of a test in administrative data was viewed as the gold standard against which self-report was validated. Only the latter three cycles of the CCHS asked respondents about history of FOBT.

We then calculated these validity measures for various demographic subgroups, specifically by self-perceived health (excellent, very good, good, fair, poor), household income ($30,000 or less, more than $30,000), highest education obtained (less than high school, high school or higher), mother tongue (English, non-English), immigrant status (Canadian-born, foreign-born), recent immigrant status among the foreign-born (immigrated 5 years or less from the date of interview vs. immigrated more than 5 years prior), employment status (currently employed vs. not), racial group (White, non-White), age category for the cervix group (24–52 years vs. 53–69 years), and gender for the FOBT group.

We calculated report-to-record ratios (the ratio of the proportion of women who reported a screening test during the relevant time period to the proportion of women who had a record of a screening test during that time period) and their confidence intervals for each screening group and for each subgroup within each screening group [23].

Finally, we conducted a multivariable regression analysis. We limited each screening group to those members who had no screening test during the appropriate time frame in administrative records, and then determined the adjusted relative risk of over-reporting (i.e. falsely reporting a screening test during that time frame), using the variables described above, with the exception of recency of immigration.

## Results

Table 1 describes the demographics of the three screening groups. All three groups were predominantly White, Canadian-born, and at least high school graduates. The majority of women (68.2%) in the cervix group were in the younger age category. In the FOBT group, 53.3% of men were employed whereas 41.5% of women were employed.

**Table 1:** **Demographic characteristics of the 39027 women in the cervical cancer screening group, 15877 women in the breast cancer screening group, and 14297 people in the FOBT screening group**

		**Cervical group**		**Breast group**		**FOBT group**		**FOBT group (male)**		**FOBT group (female)**	
	**Response**	**No.**	**%**	**No.**	**%**	**No.**	**%**	**No.**	**%**	**No.**	**%**
Gender	Male	n/a		n/a		6705	46.9	6705	100.0	n/a	
	Female	39027	100.0	15877	100.0	7592	53.1			7592	100.0
Age	24-52 years	26622	68.2	n/a		n/a		n/a		n/a	
	53-69 years	12405	31.8	n/a		n/a		n/a		n/a	
	missing		0.0								
Birthplace	Foreign born	7737	19.8	3835	24.2	3547	24.8	1679	25.0	1868	24.6
	Canadian born	31268	80.1	12030	75.8	10739	75.1	5021	74.9	5718	75.3
	missing	22	0.1	12	0.1	11	0.1	5	0.1	6	0.1
Immigrant status*	Immigrated ≤5 yrs ago	390	1.0	42	0.3	46	0.3	20	0.3	26	0.3
	Immigrated >5 yrs ago	7087	18.2	3700	23.3	3410	23.9	1613	24.1	1797	23.7
	missing	31550	80.8	12135	76.4	10841	75.8	5072	75.6	5769	76.0
Ethnicity	Non-White	3509	9.0	882	5.6	820	5.7	414	6.2	406	5.3
	White	34905	89.4	14771	93.0	13185	92.2	6148	91.7	7037	92.7
	missing	613	1.6	224	1.4	292	2.0	143	2.1	149	2.0
Education status	Less than high school	4909	12.6	3852	24.3	3546	24.8	1685	25.1	1861	24.5
	High school or higher	33939	87.0	11928	75.1	10661	74.6	4974	74.2	5687	74.9
	missing	179	0.5	97	0.6	90	0.6	46	0.7	44	0.6
Employment	Not employed	12159	31.2	8875	55.9	7557	52.9	3124	46.6	4433	58.4
	Employed	26832	68.8	6986	44.0	6720	47.0	3572	53.3	3148	41.5
	missing	36	0.1	16	0.1	20	0.1	9	0.1	11	0.1
Mother tongue	Non-English	8849	22.7	3831	24.1	3561	24.9	1707	25.5	1854	24.4
	English	30151	77.3	12035	75.8	10722	75.0	4991	74.4	5731	75.5
	missing	27	0.1	11	0.1	14	0.1	7	0.1	7	0.1
Household income	$30,000 or less	19692	50.5	9129	57.5	6606	46.2	2224	33.2	4382	57.7
	More than $30,000	16838	43.1	5197	32.7	6456	45.2	4008	59.8	2448	32.2
	missing	2497	6.4	1551	9.8	1235	8.6	473	7.1	762	10.0
Self-perceived health	Poor	1078	2.8	757	4.8	625	4.4	316	4.7	309	4.1
	Fair	3112	8.0	2066	13.0	1759	12.3	868	12.9	891	11.7
	Good	10043	25.7	4595	28.9	4242	29.7	2030	30.3	2212	29.1
	Very Good	15227	39.0	5519	34.8	5030	35.2	2317	34.6	2713	35.7
	Excellent	9555	24.5	2933	18.5	2630	18.4	1166	17.4	1464	19.3
	missing	12	0.0	7	0.0	11	0.1	8	0.1	3	0.0
Overall		39027	100.0	15877	100.0	14297	100.0	6705	100.0	7592	100.0

*Among those who were foreign-born.

The measures of validity of self-report for the three groups are reported in Table 2. Sensitivity was overall quite high for the cervix (96.5% [95% confidence interval 96.3-96.7%]) and breast (96.6% [96.3-97.0%]) groups, but was noticeably lower for the FOBT group (77.4% [75.7-79.0%]). Specificity was lowest for the cervix group (49.5% [48.6-50.5%]) and highest for the FOBT group (89.8% [89.2-90.3%]). The FOBT group had the lowest overall positive predictive value (62.1% [60.4-63.8%] and highest overall negative predictive value (94.8% [94.4-95.2%]. The highest kappa statistic value was for women in the breast group who reported excellent self-perceived health (0.677) and the lowest was for women in the cervix group who were foreign-born (0.505).

**Table 2:** **Measures of validity of self-report for cervical cancer screening, breast cancer screening, and fecal occult blood testing, using administrative data as the gold standard**

	**Sensitivity (%)**	**Specificity (%)**	**Positive predictive value (%)**	**Negative predictive value (%)**	**Kappa statistic**
**Cervix group**					
Overall	96.5 (96.3-96.7)	49.5 (48.6-50.5)	82.7 (82.3-83.1)	84.9 (84.0-85.8)	0.526
AGE					
24-52 years	96.8 (96.6-97.1)	46.6 (45.4-47.8)	84.4 (83.9-84.8)	83.1 (81.9-84.3)	0.508
53-69 years	95.6 (95.2-96.1)	54.0 (52.5-55.5)	78.7 (77.9-79.5)	87.4 (86.2-88.7)	0.542
Birthplace					
Canadian-born	97.3 (97.1-97.5)	48.5 (47.5-49.6)	82.9 (82.4-83.3)	87.7 (86.7-88.6)	0.531
Foreign-born	92.9 (92.2-93.6)	53.3 (51.3-55.3)	81.9 (80.9-82.8)	76.8 (74.8-78.8)	0.505
Immigrant Status*					
Immigrated >5 yrs ago	93.4 (92.7-94.1)	52.8 (50.7-54.9)	81.6 (80.6-82.6)	78.0 (75.9-80.1)	0.507
Immigrated ≤5 yrs ago	83.8 (79.4-88.1)	68.1 (59.6-76.7)	86.6 (82.5-90.6)	63.1 (54.6-71.7)	0.507
Ethnicity					
White	97.1 (96.9-97.3)	48.6 (47.6-49.6)	82.6 (82.2-83.1)	86.8 (85.9-87.7)	0.527
Non-white	90.7 (89.6-91.9)	57.4 (54.5-60.4)	83.2 (81.8-84.6)	72.7 (69.6-75.7)	0.512
Education Status					
High school or higher	96.8 (96.6-97.1)	47.9 (46.9-49.0)	83.8 (83.3-84.2)	84.5 (83.5-85.5)	0.520
Less than high school	93.3 (92.4-94.2)	56.6 (54.5-58.8)	74.1 (72.6-75.5)	86.4 (84.6-88.2)	0.522
Employment					
Employed	97.1 (96.9-97.4)	47.1 (45.9-48.3)	84.2 (83.7-84.6)	85.1 (83.9-86.2)	0.518
Not employed	94.8 (94.3-95.3)	53.5 (52.0-55.0)	79.1 (78.3-79.9)	84.7 (83.4-86.1)	0.528
Mother Tongue					
English	97.4 (97.1-97.6)	48.0 (47.0-49.1)	82.8 (82.3-83.2)	87.6 (86.7-88.6)	0.526
Non-English	93.4 (92.8-94.0)	54.3 (52.4-56.1)	82.3 (81.4-83.2)	78.3 (76.4-80.2)	0.522
Household Income					
More than $30,000	97.6 (97.3-97.8)	45.9 (44.4-47.5)	85.3 (84.7-85.9)	85.4 (84.0-86.9)	0.517
$30,000 or less	95.6 (95.3-96.0)	51.1 (49.9-52.3)	80.5 (79.8-81.1)	84.8 (83.6-85.9)	0.522
Self-Perceived Health					
Excellent	97.1 (96.8-97.4)	47.7 (45.7-49.7)	85.0 (84.2-85.8)	84.0 (82.1-86.0)	0.523
Very good	97.1 (96.8-97.4)	47.1 (45.5-48.6)	83.7 (83.0-84.3)	85.4 (83.9-86.9)	0.516
Good	95.5 (95.0-96.0)	51.4 (49.7-53.2)	81.3 (80.5-82.2)	83.9 (82.2-85.5)	0.525
Fair	94.9 (93.9-95.9)	54.3 (51.5-57.1)	76.8 (75.1-78.5)	86.9 (84.5-89.3)	0.529
Poor	93.8 (91.9-95.6)	55.5 (50.9-60.1)	74.3 (71.3-77.4)	86.6 (82.7-90.5)	0.518
**Breast group**					
Overall	96.6 (96.3-97.0)	64.3 (63.1-65.5)	82.1 (81.4-82.8)	91.8 (91.0-92.6)	0.649
Birthplace					
Canadian-born	96.8 (96.4-97.2)	64.9 (63.4-66.3)	82.5 (81.8-83.3)	92.1 (91.2-93.1)	0.657
Foreign-born	96.1 (95.3-96.9)	62.7 (60.2-65.1)	80.7 (79.2-82.1)	90.9 (89.1-92.7)	0.625
Immigrant Status*					
Immigrated >5 yrs ago	96.2 (95.4-97.0)	62.7 (60.2-65.3)	80.9 (79.4-82.3)	91.0 (89.2-92.8)	0.628
Immigrated ≤5 yrs ago	89.5 (75.7-100.0)	69.6 (50.8-88.4)	70.8 (52.6-89.0)	88.9 (74.4-100.0)	0.578
Ethnicity					
White	96.7 (96.3-97.1)	64.3 (63.1-65.6)	82.3 (81.5-83.0)	92.0 (91.1-92.8)	0.651
Non-white	94.6 (92.7-96.5)	62.1 (57.0-67.2)	79.7 (76.6-82.8)	88.0 (83.9-92.1)	0.599
Education status					
More than high school	96.9 (96.5-97.2)	63.4 (62.0-64.9)	83.1 (82.4-83.9)	91.6 (90.5-92.6)	0.649
High school or less	95.8 (94.9-96.6)	66.4 (64.2-68.7)	78.6 (77.0-80.1)	92.4 (90.9-93.9)	0.642
Employment					
Employed	96.6 (96.0-97.1)	63.6 (61.8-65.4)	81.5 (80.5-82.6)	91.8 (90.5-93.1)	0.641
Not employed	96.6 (96.2-97.1)	64.9 (63.2-66.5)	82.5 (81.6-83.5)	91.8 (90.7-92.9)	0.655
Mother Tongue					
English	96.7 (96.3-97.1)	64.5 (63.1-66.0)	82.5 (81.8-83.3)	91.9 (91.0-92.9)	0.654
Non-English	96.2 (95.4-97.0)	63.7 (61.2-66.1)	80.7 (79.2-82.1)	91.4 (89.7-93.1)	0.634
Household Income					
More than $30,000	97.4 (96.9-97.9)	60.4 (58.1-62.7)	83.1 (81.9-84.2)	92.1 (90.5-93.6)	0.632
$30,000 or less	96.1 (95.6-96.6)	66.3 (64.7-67.8)	81.2 (80.3-82.2)	91.8 (90.8-92.9)	0.655
Self-Perceived Health					
Excellent	97.1 (96.3-97.8)	65.9 (63.0-68.9)	84.6 (83.1-86.1)	92.2 (90.2-94.2)	0.677
Very good	96.8 (96.3-97.4)	63.3 (61.1-65.5)	84.3 (83.2-85.4)	90.8 (89.2-92.4)	0.651
Good	96.4 (95.7-97.1)	63.9 (61.6-66.1)	81.3 (80.0-82.6)	91.6 (90.1-93.2)	0.640
Fair	96.4 (95.3-97.5)	64.9 (61.8-68.0)	77.4 (75.2-79.5)	93.6 (91.7-95.5)	0.632
Poor	93.8 (91.2-96.3)	65.6 (61.0-70.2)	70.4 (66.3-74.6)	92.3 (89.3-95.4)	0.582
**FOBT group**					
Overall	77.4 (75.7-79.0)	89.8 (89.2-90.3)	62.1 (60.4-63.8)	94.8 (94.4-95.2)	0.612
Gender					
Female	77.6 (75.4-79.7)	90.7 (90.0-91.5)	66.1 (63.8-68.4)	94.5 (94.0-95.1)	0.640
Male	77.1 (74.6-79.6)	88.7 (87.8-89.5)	57.5 (55.0-60.1)	95.1 (94.5-95.7)	0.579
Birthplace					
Canadian-born	78.3 (76.4-80.2)	89.7 (89.0-90.3)	61.4 (59.5-63.4)	95.2 (94.7-95.6)	0.613
Foreign-born	74.8 (71.5-78.0)	90.0 (88.9-91.1)	64.0 (60.7-67.3)	93.7 (92.8-94.7)	0.609
Immigrant Status*					
Immigrated >5 yrs ago	75.3 (72.0-78.7)	90.1 (89.0-91.2)	64.4 (61.0-67.8)	93.9 (93.0-94.8)	0.615
Immigrated ≤5 yrs ago	54.5 (25.1-84.0)	88.6 (78.0-99.1)	60.0 (29.6-90.4)	86.1 (74.8-97.4)	0.445
Ethnicity					
White	77.4 (75.7-79.1)	89.9 (89.3-90.4)	62.3 (60.5-64.1)	94.8 (94.4-95.3)	0.615
Non-white	73.5 (66.5-80.5)	89.4 (87.1-91.7)	61.0 (53.9-68.1)	93.7 (91.8-95.6)	0.583
Education Status					
More than high school	77.7 (75.9-79.5)	89.4 (88.7-90.0)	62.9 (61.0-64.8)	94.5 (94.0-95.0)	0.615
High school or less	75.4 (71.8-79.1)	90.8 (89.8-91.8)	59.0 (55.3-62.7)	95.5 (94.7-96.2)	0.594
Employment					
Employed	76.7 (74.1-79.2)	90.7 (89.9-91.4)	61.2 (58.6-63.7)	95.3 (94.7-95.9)	0.611
Not employed	77.8 (75.7-80.0)	88.9 (88.1-89.7)	62.8 (60.5-65.0)	94.3 (93.7-94.9)	0.611
Mother Tongue					
English	79.2 (77.3-81.0)	89.4 (88.8-90.1)	61.6 (59.7-63.5)	95.2 (94.8-95.7)	0.617
Non-English	72.1 (68.7-75.5)	90.7 (89.6-91.7)	63.6 (60.1-67.0)	93.5 (92.6-94.4)	0.597
Household Income					
More than $30,000	78.3 (76.0-80.7)	89.1 (88.2-89.9)	61.6 (59.1-64.0)	94.8 (94.2-95.4)	0.610
$30,000 or less	76.0 (73.5-78.5)	90.2 (89.4-91.0)	61.8 (59.2-64.3)	94.8 (94.2-95.4)	0.607
Self-Perceived Health					
Excellent	79.4 (75.8-82.9)	89.6 (88.3-90.9)	63.7 (59.9-67.5)	94.9 (94.0-95.9)	0.630
Very good	79.2 (76.7-81.7)	89.3 (88.3-90.2)	65.0 (62.4-67.7)	94.4 (93.7-95.2)	0.633
Good	75.9 (72.8-79.1)	90.7 (89.8-91.7)	62.6 (59.4-65.8)	94.9 (94.1-95.6)	0.615
Fair	73.3 (67.8-78.8)	89.7 (88.2-91.3)	54.3 (49.0-59.6)	95.3 (94.2-96.4)	0.545
Poor	65.7 (54.6-76.8)	87.9 (85.2-90.6)	40.7 (31.6-49.8)	95.3 (93.5-97.1)	0.423

*Among those who were foreign-born.

**Figure 1:**
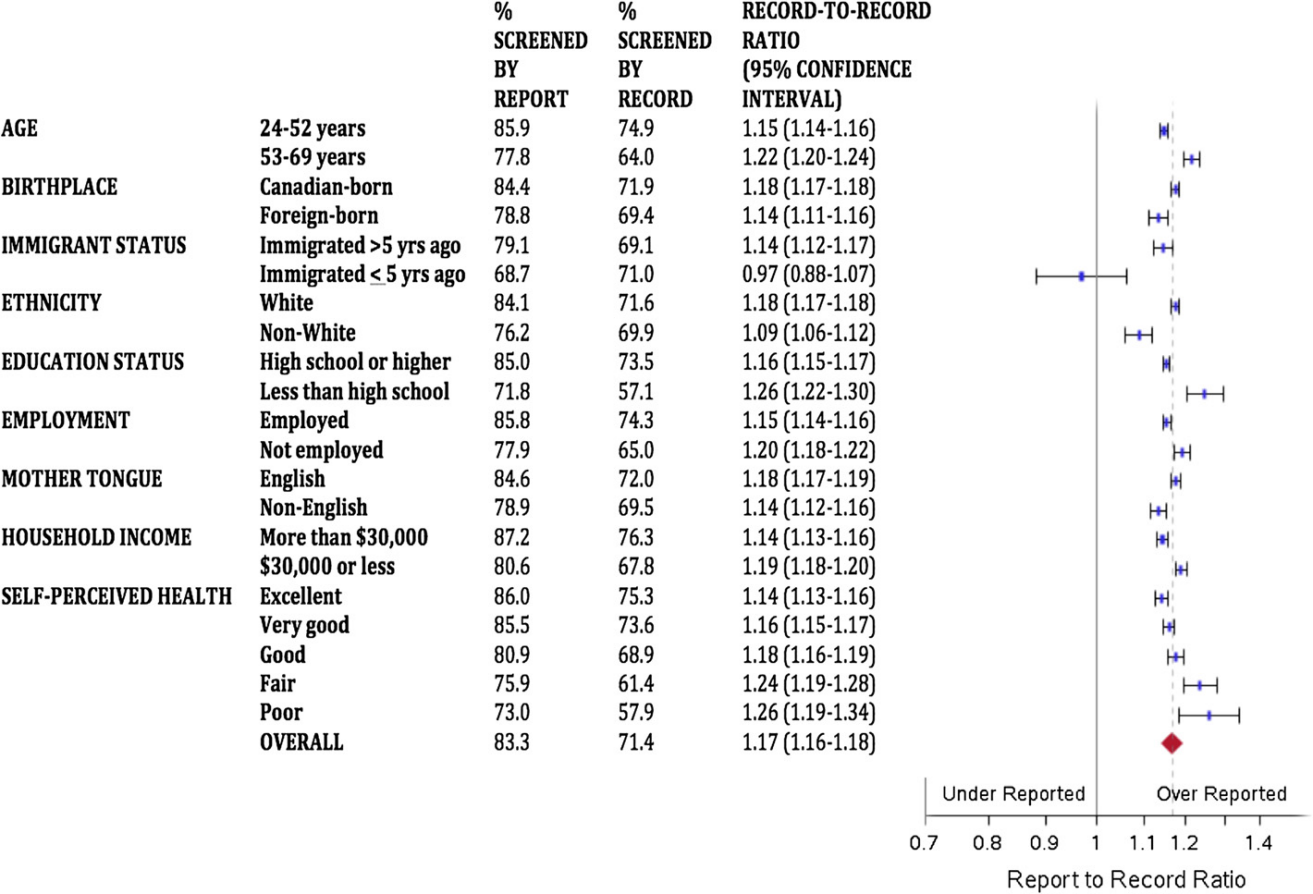
**Report-to-record ratios for cervical cancer screening.**

**Figure 2:**
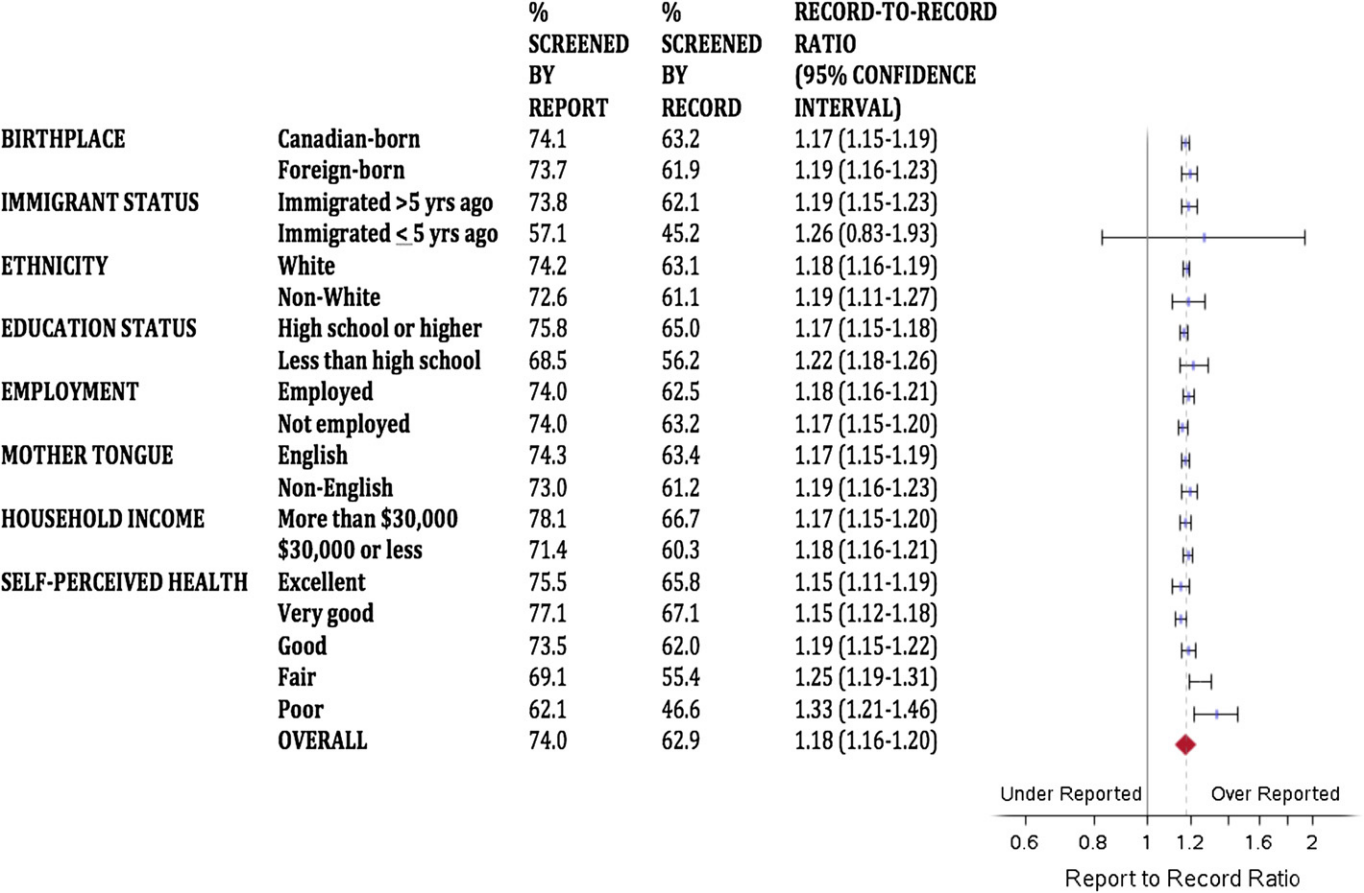
**Report-to-record ratios for breast cancer screening.**

**Figure 3:**
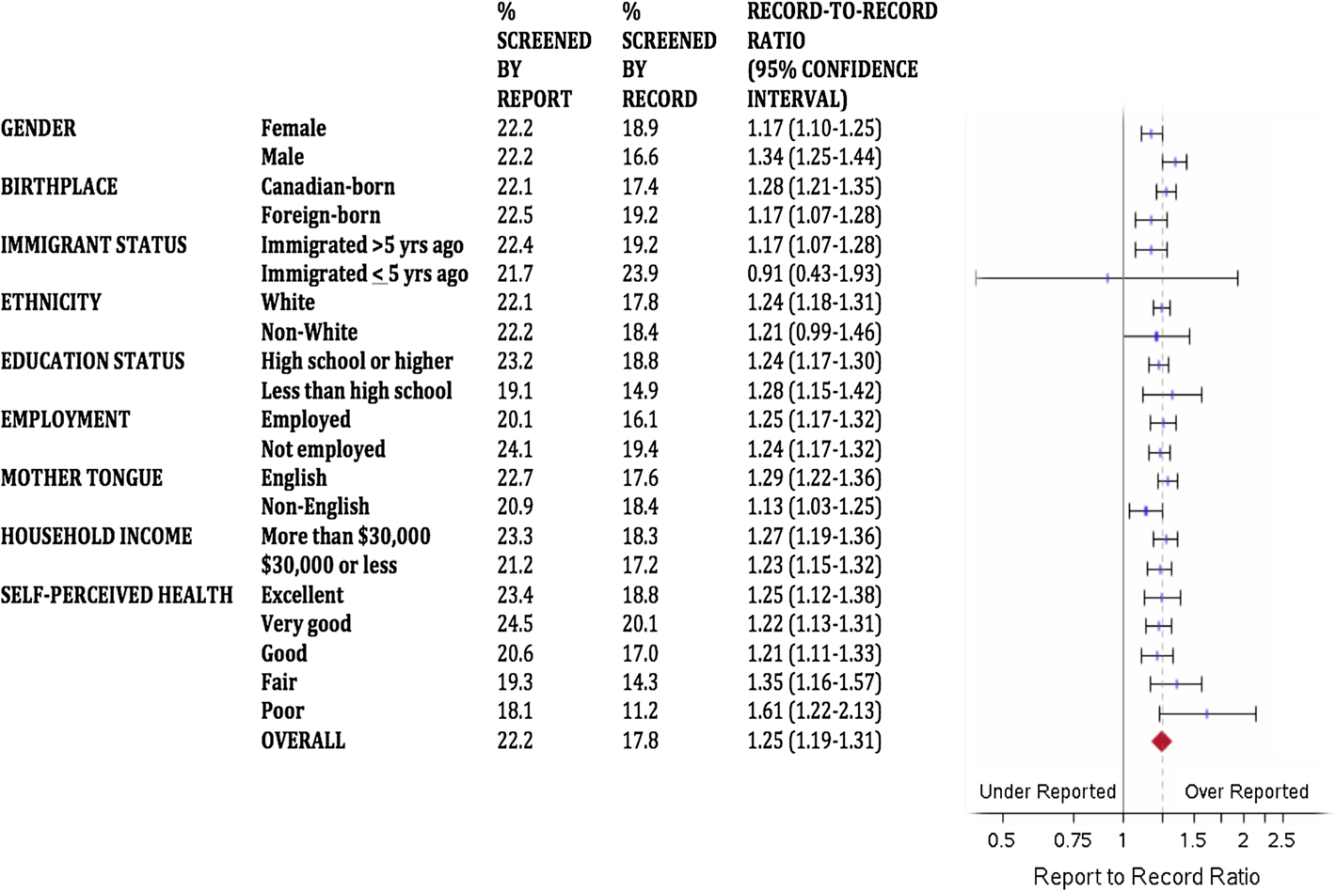
**Report-to-record ratios for fecal occult blood testing.**

Figures 1, 2 and 3 describe the report-to-record ratios for each screening group and demographic subgroup. Ratios were persistently significantly greater than 1, suggesting pervasive over-reporting, except for more recent immigrants where confidence intervals crossed unity for each screening type. For the cervix group (Figure 1), women who were older, less educated, and of fair or poor self-perceived health had report-to-record ratios that were higher than the overall estimate, and women who were younger, foreign-born, recent immigrants, non-White, and non-English speaking had ratios lower than the overall estimate. For the breast group (Figure 2), women of poor self-perceived health had a report-to-record ratio higher than the range of the overall estimate. For the FOBT group (Figure 3), no group had a report-to-record ratio outside of the range of the overall estimate. The FOBT group (Figure 3) had the highest overall report-to-record ratio (1.246 [1.189-1.306]) of the three groups.

In multivariable regression analyses, women who were younger, White, higher educated, English native speakers, and of higher income were less likely to over-report cervical cancer screening. Higher income women were less likely to over-report breast cancer screening. Men were less likely to over-report FOBT use, whereas employed people were more likely to over-report FOBT use (adjusted relative risk 1.18 [95% confidence interval 1.03-1.36] (Table 3)).

**Table 3:** **Adjusted relative risks [with 95% confidence intervals] of over-reporting, defined as reporting a Pap test in the past 3 years (cervix group), a mammogram in the past 2 years (breast group), and a fecal occult blood test in the past 2 years (FOBT group), but with no record of same in administrative data**

		**Cervix group**	**Breast group**	**FOBT group**
Age	24-52 years vs. 53–69 years	0.88 [0.84-0.92]*	n/a	n/a
Gender	Male vs. female	n/a	n/a	0.81 [0.71-0.92]*
Birthplace	Canadian-born vs. foreign-born	0.99 [0.94-1.06]	1.06 [0.96-1.16]	1.00 [0.84-1.19]
Ethnicity	White vs. non-White	0.85 [0.78-0.92]*	1.00 [0.85-1.18]	1.17 [0.90-1.53]
Education Status	High school or higher vs. less than high school	0.89 [0.84-0.94]*	0.95 [0.87-1.03]	0.89 [0.76-1.05]
Employment	Employed vs. not employed	0.98 [0.94-1.03]	1.02 [0.94-1.10]	1.18 [1.03-1.36]*
Mother Tongue	English vs. non-English	0.92 [0.87-0.97]*	1.00 [0.91-1.10]	0.91 [0.77-1.08]
Household Income	More than $30,000 vs. $30,000 or less	0.96 [0.92-1.00]*	0.84 [0.77-0.91]*	0.89 [0.77-1.03]
Self-Perceived Health	Excellent vs. poor	1.02 [0.97-1.07]	1.10 [0.99-1.23]	0.99 [0.83-1.17]
	Very good vs. poor	0.97 [0.92-1.02]	1.09 [0.97-1.22]	0.84 [0.69-1.01]
	Good vs. poor	0.94 [0.87-1.02]	1.07 [0.94-1.23]	0.90 [0.71-1.14]
	Fair vs. poor	0.93 [0.82-1.04]	1.08 [0.91-1.29]	1.13 [0.83-1.54]

Adjusted for variables listed in table.

*Statistically significant result.

## Discussion

In this study of the validity of self-report of up-to-date cancer screening among Ontarians, using administrative health data as our gold standard, we found that there is cause for concern when using self-report as the sole method of quantifying cancer screening rates. Specificity was less than 70% overall and for all subgroups for cervical and breast cancer screening and sensitivity was lower than 80% overall and for all subgroups for screening with FOBT. Report-to-record ratios were pervasively significantly greater than 1, indicating that over-reporting of cancer screening is common in Ontario.

Self-report of FOBT stood out among the three cancer screening types for having the highest report-to-record ratios, the lowest sensitivity and positive predictive values, and the highest specificity and negative predictive values. Taken together, these results suggest that false positives and true negatives are both quite high for FOBT use, with true negatives predominating. The high false positive rate implies that estimates of FOBT utilization based on self-report could be particularly erroneous and should be avoided where possible, and our finding of high true negatives is in line with the known low prevalence of CRC screening by FOBT in Ontario [24].

There was no clear pattern suggesting that Ontarians with social disadvantage were consistently more likely to over-report than their more advantaged peers. For example, in regression analyses, women who were White, higher educated, English native speakers and of higher income were less likely to over-report cervical cancer screening in Ontario than their respective counterparts, but this did not hold true for the other screening types. The higher sample size in the cervix group may have driven the higher number of significant results for cervical cancer screening than for the other two forms of screening. However, we have demonstrated that sociodemographic characteristics do appear to play a role in Ontario populations and should be considered when examining cancer screening gaps based on self-reported data.

Report-to-record ratios indicated that people of poorer self-rated health tended to be more likely to over-report up-to-date screening than those of better self-rated health for both cervical and breast cancer screening. The reason for this finding is unclear, but it has been reported elsewhere in the literature [25]. It may reflect more frequent contact with the health care system for people of poorer self-rated health, and therefore a more difficult time differentiating which medical tests and procedures one has received. Distinct event are easier for patients to remember, and screening may be less likely to be a distinct event for patients of worse health status [26].

Our findings have implications for public health researchers, practitioners and policymakers who are interested in cancer screening in Ontario. It is apparent that self-report of cancer screening is not the most accurate way to determine cervical, breast and colorectal cancer screening rates in Ontario, and will over-estimate screening rates. Where possible, the use of more objective data should be encouraged. Where not possible, the pervasive over-reporting that we demonstrated should be remembered and considered. The results we have presented could be used in the Ontario or Canadian setting as correction factors to determine more accurate screening rates [27]. Also, our findings suggest that there are opportunities to increase patients’ basic knowledge about screening, and their understanding of which screening tests they have and have not received. Many women mistake any pelvic examination for a Pap test [28-30], and it is feasible that patients may similarly mistake digital rectal exams for FOBT screening [31]. Better provider-patient communication and patient education may improve the accuracy of self-report, and may be of particular importance for disadvantaged populations [14,28].

Few Canadian studies have examined the validity of self-report of cancer screening for cervical, breast, and colorectal cancer. In their 2009 study, Wang et al. demonstrated that rates in Ontario administrative data were consistently lower for breast and cervical cancer screening and for influenza vaccination than rates in self-reported CCHS data [32]. Khoja et al. compared self-report of any form of CRC screening to medical records from physicians’ offices and found a kappa of 0.66. They reported that it was common for study participants to believe a test had occurred more recently than records showed [33]. Fehringer et al. compared cervical cancer screening rates in Cytobase, Ontario’s Pap test registry, with self-reported CCHS rates across 37 public health units in the province and found that Cytobase rates were consistently lower, concluding this was likely due to over-reporting [34]. Walker et al. found that women at high familial risk of breast cancer accurately reported mammogram use within the previous 12 months [35]; however, Larouche et al. assessed validity of self-reported mammography among women who had undergone breast cancer-specific genetic testing and concluded that self-report overestimates mammography use in this population [36].

This study has several limitations. First, administrative data are not a perfect gold standard [13,32]. For example, Pap tests performed and interpreted solely within hospitals in Ontario may not be captured by either administrative data or by Cytobase. However, the number of screening Pap tests to which this would apply is expected to be quite small. As well, we used a variety of databases (OHIP, OBSP, Cytobase) to find objective records of screening. Second, the CCHS is voluntary. Therefore, CCHS respondents, particularly those who agreed to have their survey answers linked to administrative health data, may systematically differ from the general population. However, they are likely to be representative of any sample who would agree to self-report cancer screening. Third, the CCHS has some missing data, particularly around income levels. Although these missing data affect the power of our results, they are unlikely to affect the veracity of our findings. Finally, we dichotomized many of the characteristics that we examined, such as race, recent immigrant status, and education, which may not have provided enough detail to uncover differences. For example, ethnicity has been reported as a factor affecting self-report in the US literature, with African-Americans and Hispanics highlighted [14,15]. Although we did examine racial group in this study, we only looked at White vs. non-White. However, sample size often would not allow for more detailed categorization.

## Conclusions

In this work, we have found that in Ontario as in other jurisdictions, there is a pervasive tendency for people to over-report their cancer screening histories. Sociodemographic status also appears to influence the likelihood of over-reporting screening. Public health practitioners and policymakers need to be aware of the limitations of self-report and adjust their methods and interpretations accordingly.
